# Novel 1,2,3-Triazole-Coumarin Hybrid Glycosides and Their Tetrazolyl Analogues: Design, Anticancer Evaluation and Molecular Docking Targeting EGFR, VEGFR-2 and CDK-2

**DOI:** 10.3390/molecules27072047

**Published:** 2022-03-22

**Authors:** Wael A. El-Sayed, Fahad M. Alminderej, Marwa M. Mounier, Eman S. Nossier, Sayed M. Saleh, Asmaa F. Kassem

**Affiliations:** 1 Department of Chemistry, College of Science, Qassim University, Buraidah 51452, Saudi Arabia; w.shendy@qu.edu.sa (W.A.E.-S.); e.saleh@qu.edu.sa (S.M.S.); 2 Photochemistry Department, National Research Centre, Dokki, Giza 12622, Egypt; 3 Department of Pharmacognosy, National Research Centre, Giza 12622, Egypt; marwa_m3@yahoo.com; 4 Department of Pharmaceutical Medicinal Chemistry and Drug Design, Faculty of Pharmacy (Girls), Al-Azhar University, Cairo 11754, Egypt; dr.emannossier@gmail.com; 5 Chemistry Branch, Department of Science and Mathematics, Faculty of Petroleum and Mining Engineering, Suez University, Suez 43721, Egypt; 6 Department of Chemistry of Natural and Microbial Products, National Research Centre, Cairo 12622, Egypt

**Keywords:** 1,2,3-triazoles, coumarin, tetrazoles, anticancer, glycosides, molecular docking, EGFR, CDK-2

## Abstract

This study represents the design and synthesis of a new set of triazole-coumarin-glycosyl hybrids and their tetrazole hybrid analogues possessing various sugar moieties and modified analogues. All the newly synthesized derivatives were screened for their cytotoxic activities against a panel of human cancer cell lines. The coumarin derivatives **10**, **13** and **15** derivatives revealed potent cytotoxic activities against Paca-2, Mel-501, PC-3 and A-375 cancer cell lines. These promising analogues were further examined for their inhibitory assessment against EGFR, VEGFR-2 and CDK-2/cyclin A2 kinases. The coumarin-tetrazole **10** displayed broad superior inhibitory activity against all screened enzymes compared with the reference drugs, erlotinib, sorafenib and roscovitine, respectively. The impact of coumarin-tetrazole **10** upon cell cycle and apoptosis induction was determined to detect its mechanism of action. Additionally, it upregulated the levels of casp-3, casp-7 and cytochrome-c proteins and downregulated the PD-1 level. Finally, molecular docking study was simulated to afford better rationalization and gain insight into the binding affinity between the promising derivatives and their targeted enzymes, which could be used as an optimum lead for further modification in the anticancer field.

## 1. Introduction

Cancer is one of the most serious and complicated health disorders all over the world [1]. Owing to the incidence of resistance, toxicity, and lack of selectivity of the currently existing anticancer drugs, targeted therapies have recently been approved for the treatment of definite cancers such as lung, breast, pancreatic and colorectal cancers as well as lymphoma and leukaemia [2].

Several kinases function as an on/off switch for cellular motility and proliferation. Thus, mutations of these kinases are responsible for cellular irregularities affording cancer initiation, metastasis or progression [3]. Multiple kinase inhibitors are now applied as a beneficial strategy in targeted therapies for the treatment of human malignancies [4].

One of the most prominent oncogenic kinases is epidermal growth factor receptor (EGFR), which is a receptor tyrosine kinase and plays an essential mediating role in cell proliferation, angiogenesis, apoptosis and metastatic spread [5]. It is overexpressed in a multiplicity of human cancers, including breast, colorectal, lung, prostate, ovary and pancreatic cancers [6,7]. Poor treatment outcomes as a result of resistance to radiotherapy, hormone therapy, and cytotoxic drugs presented an opportunity for anti-EGFR drug recommendations due to their higher safety and efficacy compared to standard chemotherapy [5,8,9]. Moreover, vascular endothelial growth factor receptor-2 (VEGFR-2) has a crucial role in the induction of angiogenesis [10,11,12], which is correlated with tumour growth, invasion and metastasis. VEGFR-2 inhibitors are considered as interesting therapeutic targets for suppressing tumour angiogenesis and the subsequent tumour growth [13]. Additionally, cyclin-dependent kinase-2 (CDK-2) is known as cell division kinase and belongs to the Serine/Threonine kinase family. CDK-2 occupies a major part in cell cycle regulation including cell cycle propagation, differentiation, neuronal function and apoptosis [14,15]. Its overexpression will lead to several types of malignancies such as breast, lung, colorectal, ovarian and pancreatic [16]. The inhibitors of CDK-2 prompt apoptosis by disrupting the cell cycle and reflect their importance in drug discovery [17].

It has been apparent that unresponsive cancer cases that require integration therapy [18] involving the incorporation of more than one drug usually result in drug–drug interactions and eventually severe side effects. Accordingly, to conform various pharmacophores in a single candidate drug with minimum side effects, the strategy of molecular hybridization is an indispensable approach.

**Figure 1:**
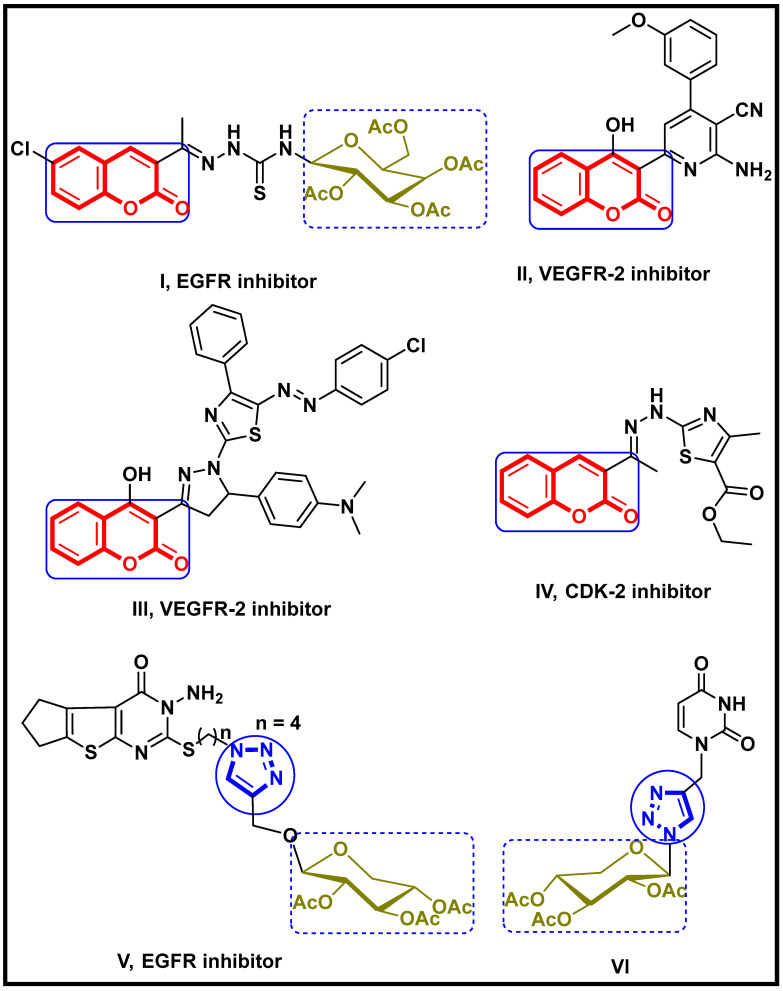
Reported anticancer agents owing coumarin, triazole and/or glycoside scaffolds with different mechanisms.

A careful audit of the literature revealed the significance of heterocyclic compounds as promising leads possessing potent anticancer activity [19,20,21,22,23,24,25]. As one of the peerless scaffolds, coumarin and its incorporating derivatives exhibited a broad spectrum of pharmacological effects such as antibacterial [26], antioxidant [27], anti-HIV and anticancer [28] activity. Coumarin derivatives **I**–**IV** have been well reported and characterized by their potent anticancer activity through inhibition of EGFR, VEGFR-2 or CDK-2 kinases [29,30,31,32] (Figure 1).

1,2,3-Triazole motifs are known to be present in a large number of compounds exhibiting anticancer [33] and anti-HIV [34,35] activity in addition to their ability to be potent pharmacophores [36,37]. Compounds possessing triazole scaffold **V** and **VI** revealed remarkable in vitro cytotoxicity against different human cancer cell lines via a significant inhibition of EGFR [8] (Figure 1). Furthermore, tetrazole, being a possible bioisoster of 1,2,3-triazole with a five-membered heterocyclic structure, has been exhibited by its derivatives anticancer [38,39], antibacterial [40,41], antifungal [42,43], antimalarial [44,45], antitubercular [46,47] activities. Various tetrazole-based drugs, e.g., Irbesartan, Cefamandole and Cilostazol, have already been used in clinical practice for the treatment of different diseases, revealing the therapeutic significance of the tetrazole scaffold [48]. It has also been revealed that the hybridization of tetrazole and pharmacophores possessing anticancer activity could perform an efficient approach to evolve new anticancer candidates [49].

On the other hand, heterocycle-glycoside hybrids are crucial systems that have acquired considerable biological interest. Compounds consisting of glycosyl heterocyclic motifs have demonstrated anticancer activity, e.g., **I**, **V** and **VI**, as well as other important biological roles such as antiviral and antimicrobial activities [50,51,52,53,54,55]. It has also been reported that coumarin-glycoside **I** and triazole-glycoside **V** displays promising anticancer and kinase inhibitory activity against EGFR [8,29] (Figure 1).

**Figure 2:**
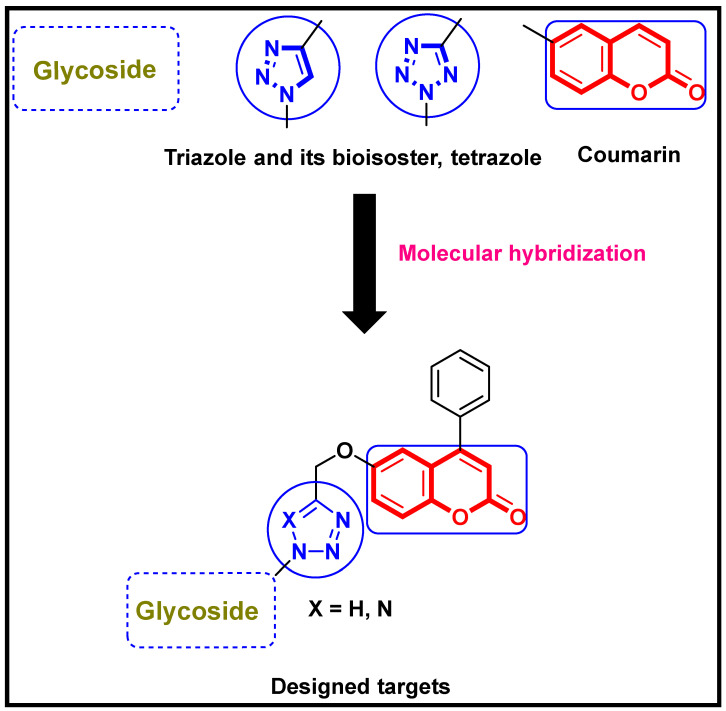
Rational of new designed coumarin-triazole or tetrazole hybrids incorporated with different glycosides targeting EGFR, VEGFR-2 and CDK-2 kinases.

In the present work, consideration has been given to the above facts and to the continuation of our research interest in finding selective and robust heterocyclic glycosylation hybrids incorporating modified hetero-base and/or glycone moieties [56,57,58,59]. So, in attempt to discover potent anticancer agents, a new set of triazole-coumarine-glycosyl hybrids and their tetrazole hybrid analogues possessing different sugar parts was designed and synthesized through molecular hybridization (Figure 2). All the newly synthesized derivatives were evaluated for their antiproliferative activity against a panel of human cancer cell lines. The promising derivatives were further investigated for their inhibitory assessment against EGFR, VEGFR-2 and CDK-2/cyclin A2 kinases, and for their impact upon cell cycle and apoptosis to detect their mechanism of action. Then, molecular docking was carried out to find their binding affinity and mode of interactions with the key amino acids of the screened enzymes. This might be used as an optimum lead for further modification.

## 2. Results and Discussion

### 2.1. Chemistry

**Scheme 1:**
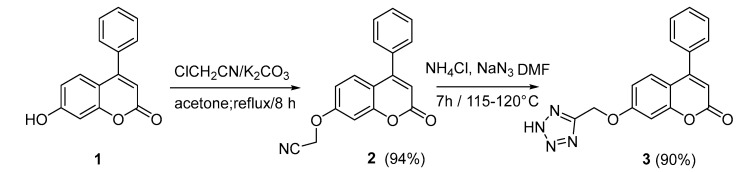
Synthesis of tetrazolylmethyloxy (coumarine) derivative.

In the current study, two new targeted glycosyl azolyl heterocycles linked to a substituted coumarin scaffold as hybrid glycoconjugates were synthesized via the Cu-catalysed dipolar cycloaddition approach or dipolar cycloaddition strategy, followed by glycosylation. The first afforded system is the glycosyl-tetrazole-based coumarin motif as a structural analogue of the prepared triazole glycosides. The second is a glycosyl-1,2,3-triazole-based coumarin motif, in which the carbohydrate moiety is directly attached to the triazole system via a C-*N*^1^ linkage. The starting hydroxycoumarin compound **1** was reacted with chloroacetonitrile and afforded the (chromen-7-yl)oxy)acetonitrile derivative **2** in 92% yield. The IR spectrum of the formed nitrile compound showed the distinctive absorption band characterizing the CN group and its ^1^H NMR spectrum revealed the singlet signal of the CH_2_ group in addition to the proton signals of the phenyl and chromenone system. The latter nitrile-based chromeneone compound **2** was reacted with sodium azide via a cycloaddition process and resulted in the formation of the tetrazole linked to chromeneone product **3** (Scheme 1).

**Scheme 2:**
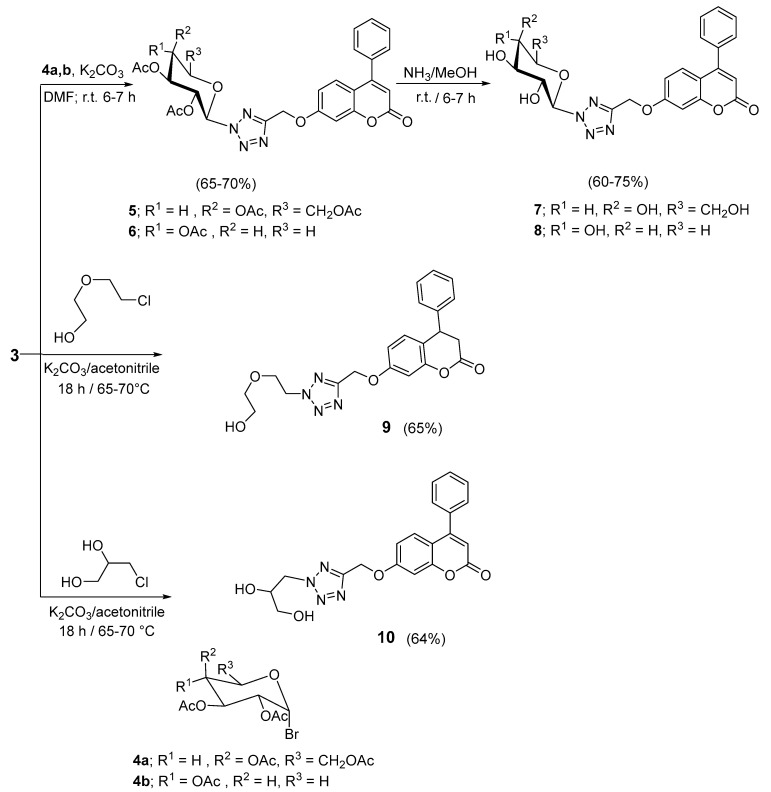
Synthesis of tetrazole glycosides and acyclic analogues.

Glycosylation of the latter produced tetrazole derivative via reaction with per-*O*-acetylated bromoglycopyranose, namely 2,3,4,6-tetra-*O*-acetyl-d-galacto- or 2,3,4-tri-*O*-acetyl-d-xylopyranosyl bromide in a basic medium, leading to the formation of the acetylated-*N*-glycosyl tetrazole derivatives **5a**,**b**, respectively. The IR spectra of the afforded tetrazole glycosides exhibited the absorption bands of the OAc carbonyl groups at their characteristic regions. The latter groups have also been revealed in the ^1^H NMR spectra in the form of the acetyl methyl proton signals and in their corresponding ^13^C NMR as the carbonyl and the methyl signals at their expected assignments. The coupling constant value of the anomeric hydrogen (H-1 in the glycopyranose sugar moiety) indicated the *β*-conformation of the attachment of the sugar part via ^1^C-N linkage. The spectra also showed the signals attributed to the remaining hydrogens in the described structures, including the CH_2_, the phenyl and the chromenone system at their known assignments (see exp. part). For the formation of the tetrazole-chromenone glycoside hybrids, the protected compounds **5a**,**b** were deacetylated by means of methanolic gaseous ammonia solution and gave the derived products **6a**,**b**, incorporating the sugar moieties with free hydroxyl groups (Scheme 2).

The key tetrazole compound **3** was also used as a substrate for the preparation of acyclic analogues of the tetrazole-*N*-glycosides via its reaction with acyclic oxygenated and hydroxylated alkyl halides. Thus, reaction of **3** with 2-(2-chloroethoxy)ethan-1-ol and 3-chloropropane-1,2-diol afforded the tetrazole-*N*-acyclic nucleoside analogues **9** and **10**, respectively (Scheme 2). Their ^1^H NMR spectra of the latter formed products exhibited the signals of the hydrogens in the attached side chain at the tetrazole nitrogen at their expected values in addition to the residual proton signals of the assigned structures. Moreover, the characteristic bands corresponding to the hydroxyl frequencies were revealed in their IR spectra in addition to the disappearance of the tetrazole NH, confirming the structures.

The second type of hybrid structures in the current study is the chromeneone bridged 1,2,3-triazole glycoside compounds as the desired analogues, incorporating the latter scaffolds exhibiting the 1,2,3-triazole core linked to a five and six-carbon sugar moiety. In such a strategy, the required terminal acetylenic substrate **11** was prepared via reaction of the starting hydroxychromenone derivative **1** with propargyl bromide, according to a previously known method [60].

The resulting terminal substituted acetylene product was reacted with a series of azido-glycopyranosyl derivatives; specifically, tetra-*O*-acetyl-*β*-d-gluco-, tetra-*O*-acetyl-*β*-d-galacto- and tri-*O*-acetyl-*β*-d-xylopyranosyl azides (**12a**–**c**), via applying the conditions of click dipolar cycloaddition, which resulted in the desired 1,2,3-glycosyl-chromenone hybrid glycosides **13**–1**5**, respectively, in 65–77% yields. For the regioselective cycloaddition reaction, the generation of the essential Cu^1^ catalytic species was achieved by incorporating sodium ascorbate in the experiment together with copper sulphate, via in situ reduction of copper (II) ions. The best solvent system that has been revealed for the reaction is butanol/water mixture (2:1) in addition to the use of DIPEA in catalytic drops. The IR spectra of formed triazolyl-*N-*glycosides **13**–**15** showed that the bands of the stretching frequencies were attributed to the acetate C=O groups at 1751–1741 cm^−1^. Their ^1^H NMR spectra showed that the signals assigned for the sugar moiety hydrogens represented the protons of the acetyl-methyl at *δ* 1.94–2.01 ppm and the assigned signals corresponded to the remaining hydrogens of the sugar part, in addition to the substituted chromenone system, according to the assigned structures. The *J*-value of the H-1 (known as the anomeric proton) equalling 9.8–10.2 Hz indicated the *β*-conformation of the mode of attachment of the glycosyl moiety linked to the triazole ring. The ^13^C NMR spectra for the ^1^*N*-1,2,3-triazole glycosides possessed the signals at δ 20.1–21.1 ppm assigned for the carbons of the methyl groups of the *O*-acetyl-sugar moiety. 

**Scheme 3:**
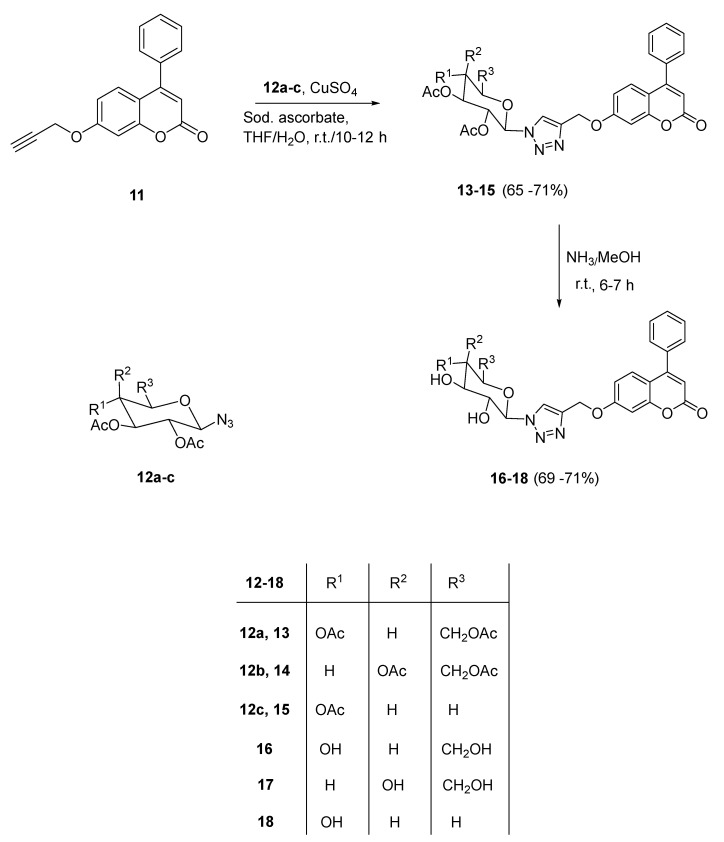
Synthesis of 1,2,3-triazole glycosides based substituted coumarin system.

Deprotection of the *O*-acetylated glycosyl-1,2,3-triazoles **13**–**15** was achieved by means of dry methanol saturated with gaseous ammonia and resulted in corresponding glycosides **16**–**18**, respectively (Scheme 3), with sugar moieties possessing the free hydroxyl groups. The evanescence of the carbonyl absorption bands in the IR spectra of the latter products and the existence of the hydroxyl characteristic absorptions at 3448–3421 cm^−1^ indicated the deacetylation process and confirmed their formation. The presence of the signals attributed to the hydroxyl groups at δ 4.45–5.20 ppm and the absence of those attributed to the glycosyl methyl signals in the corresponding ^1^H-NMR spectra confirmed the assigned structures incorporating the deacetylated sugar moieties.

### 2.2. Biological Evaluation

#### 2.2.1. In Vitro Cytotoxicity

**Figure 3:**
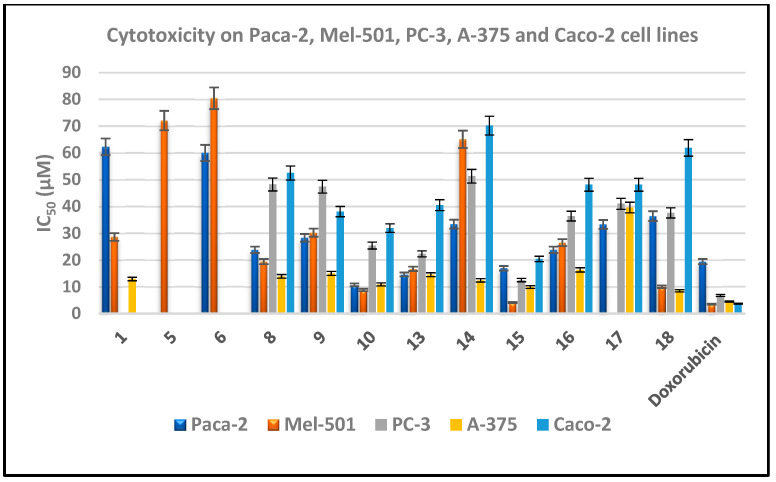
Cytotoxicity of the promising synthesized targets against human cancer Paca-2, Mel-501, PC-3, A-375 and Caco-2 cell lines through the MTT assay.

The cytotoxic screening of all synthesized derivatives **1–18** was estimated against human pancreatic (Paca-2), melanoma (Mel-501), prostate (PC-3), melanoma (A-375) and colon (Caco-2) cancer cell lines through the colorimetric MTT assay [61,62,63], compared with the reference drug, doxorubicin at a concentration of 100 μM (Table 1). The promising derivatives that afforded cytotoxic activity ≥ 60% at a concentration of 100 μM were further screened against their corresponding cells in a dose-dependent manner at concentrations ranging from (100 to 0.78 μM) to calculate their IC_50_, as shown in Table 2 and Figure 3. By inspection of the gained data, it was observed that the synthesized derivatives **10**, **13** and **15** displayed the highest and promising cytotoxic activity against Paca-2, Mel-501, PC-3 and A-375 cancer cell lines, while the promising activity of compound **18** appeared against Mel-501 and A-375 cells only. Additionally, it was noted that the pancreatic Paca-2 was the most sensitive cancer cell line for the promising cytotoxic derivatives. On the other hand, the observed results revealed that the newly synthesized derivatives gave weak cytotoxicity with Caco-2 cancer cell lines compared with the reference (IC_50_ range 20.4–70.2 µM and IC_50 doxorubicin_ = 3.7 µM). 

##### Structure Activity Relationship Study

Coumarin-tetrazole derivatives afforded weak to moderate anticancer activity either without glycoside fragments as in compound **3** or with acetylated glycosides as in compounds **5** and **6** or hydroxylated glycosides as in compounds **7** and **8**. Insertion of 2-hydroxyethyl fragment at N-2 of tetrazole ring in compound **9** exhibited moderate activity against Paca-2 and A-375 cell lines (IC_50_ = 28.3 and 15 µM, respectively) in comparison with the standard (IC_50_ = 19.4 and 4.5 µM, respectively). Replacing with 2,3-dihydroxypropyl fragment in compound **10** showed excellent potency (IC_50_ = 10.7, 8.8 and 10.9 µM against Paca-2, Mel-501 and A-375 cell lines, respectively). 

On the other hand, the coumarin-triazole scaffold illustrated cytotoxicity ranged from moderate to excellent **13**–**18**. The derivatives bearing acetylated glycosides revealed excellent potency, except compound **14**, while the hydroxylated ones gave moderate activity.

Finally, it was concluded that excellent potency was established through the incorporation of either coumarin-tetrazole scaffold with 2,3-dihydroxypropyl fragment or coumarin-triazole with acetylated glycosides.

#### 2.2.2. In Vitro Enzyme Inhibitory Assessment against EGFR, VEGFR-2 and CDK-2

In order to identify the possible anticancer mechanisms of the promising synthesized targets **10**, **13** and **15**, kinase profiling assessment was estimated against EGFR, VEGFR-2 and CDK-2/cyclin A2 using erlotinib, sorafenib and roscovitine as standard drugs, respectively [5,64,65]. The kinase inhibitory data are depicted as IC_50_ values (μM) in Table 3.

In view of the obtained data, it was noticed that coumarin-tetrazole **10** afforded superior inhibitory activity against EGFR, VEGFR-2 and CDK-2/cyclin A2 (IC_50_ = 0.09 ± 0.21, 0.86 ± 0.25 and 0.19 ± 0.05 μM, respectively) compared with the reference drugs, erlotinib, sorafenib and roscovitine (IC_50_ = 0.13 ± 0.020, 1.050 ± 0.20 and 0.37 ± 0.22 μM, respectively). Moreover, the coumarin-triazoles **13** and **15** displayed weak activities against EGFR and VEGFR-2 (IC_50_ = 66 ± 0.01, 53 ± 0.15 μM against EGFR and 45 ± 0.10, 40 ± 0.05 μM against VEGFR-2, respectively) and excellent activity against CDK-2/cyclin A2 (IC_50_ = 0.50 ± 0.11 and 0.48 ± 0.15 µM, respectively).

The obtained data illustrated that the coumarin-tetrazole **10** could be considered as multi-targeted kinase inhibitor against EGFR, VEGFR-2 and CDK-2/cyclinA2, while the coumarin-triazoles **13** and **15** demonstrated cytotoxicity through the inhibition of CDK-2.

#### 2.2.3. Cell Cycle Arrest and Apoptosis of Compound **10**

Programmed cell death has been targeted as a chemotherapeutic approach to fight cancer. There are specific patterns associated with programmed cell death, including nuclear fragmentation and cell shrinkage. Extrinsic and intrinsic pathways are the two major apoptotic pathways involved in mitochondrial pathways. It is known that mitochondria play a vital role in many biosynthetic processes, along with their role in apoptotic cell death [66].

**Figure 4:**
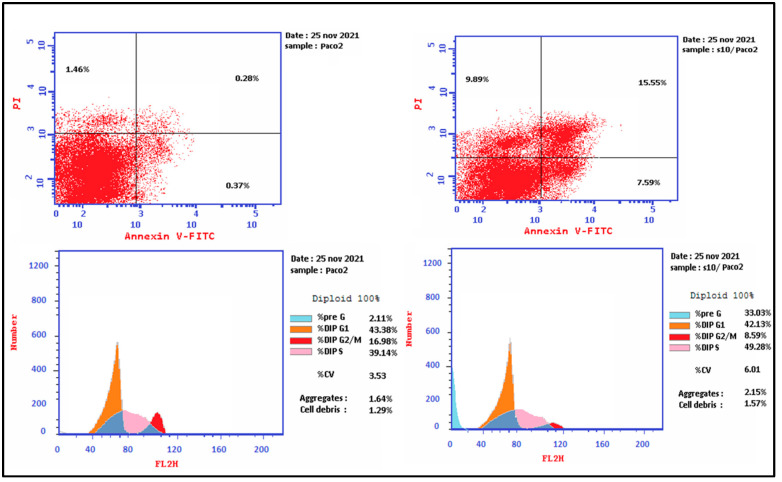
Cell cycle analysis and the effect of the target **10** on the percentage of V-FITC-positive annexin staining in Paca-2 cells comparing with the control.

**Figure 5:**
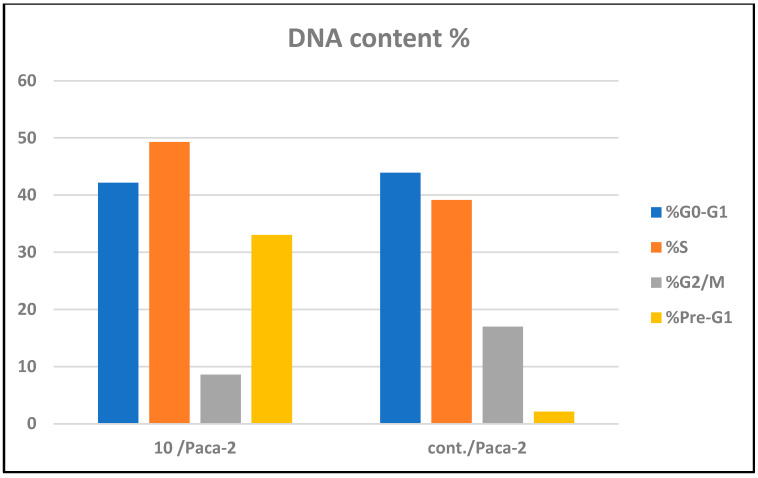
Cell cycle analysis of the coumarin-tetrazole derivative **10**.

**Figure 6:**
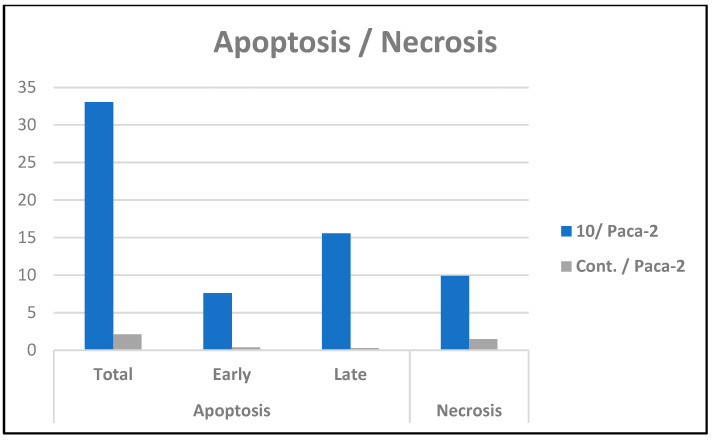
Apoptotic activity of the coumarin-tetrazole derivative **10**.

As a further investigation, Paca-2 cells were subjected to the derivative **10** for 24 h at its IC_50_ value (10.7 μM) to explore its impact on the cell cycle profile and apoptosis induction using flow cytometry (Figure 4, Figure 5 and Figure 6). The exposure of Paca-2 cells to the compound **10** affected the normal cell cycle sequence by increasing the pre-G1 phase cells percentage by 15.7 folds and the percentage of the cells at S phase by 1.3 folds, compared to the control. Accumulation of cells in pre-G1 phase is expected to be caused by the genetic materials fragmentation or degradation highlighting the role of compound **10** in apoptosis and induction of cytotoxicity. Furthermore, the arrest of cell cycle at S phase might reflect the ability of the compound **10** to disrupt DNA replication and prevention of further cell proliferation. By focusing upon apoptosis, there was a reduction in cell survival percentage and a noticeable increase in the late/secondary cellular apoptosis from 0.28% (DMSO control) to 15.55% for the screened compound, Figure 6.

#### 2.2.4. Effect of Compound **10** on the Levels of Caspase-3, 7, Cytochrome-c and PD1 Protein in Paca-2

The Mitochondria apoptotic pathway involves mitochondrial outer membrane permeabilization or MOMP, followed by cytochrome-c release into the cytosol, leading to the activation of pro-caspase-9, which in turn cleaves and activates the executioner caspases-3 and -7 [67]. The executioner caspase is known by its ability to effectively kill the cell within minutes through the parallel cleavage of hundreds of different substrates. Furthermore, immunotherapy combined with chemotherapy has generated increasing interest in cancer research. The main objective of immunotherapy is to use the body’s immune system to attack cancer cells. A PD-1 inhibitor is a powerful group that activates the immune system to attack tumours and is used to treat certain types of cancer. In this manner, the most active compound **10** was chosen to study its effect on certain proteins as casp-3, casp-7, PD-1 and cytochrome-c.

**Figure 7:**
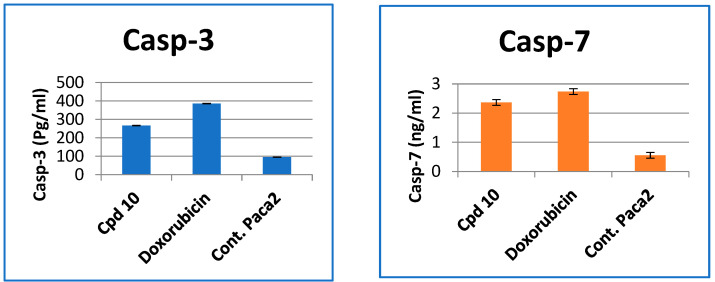

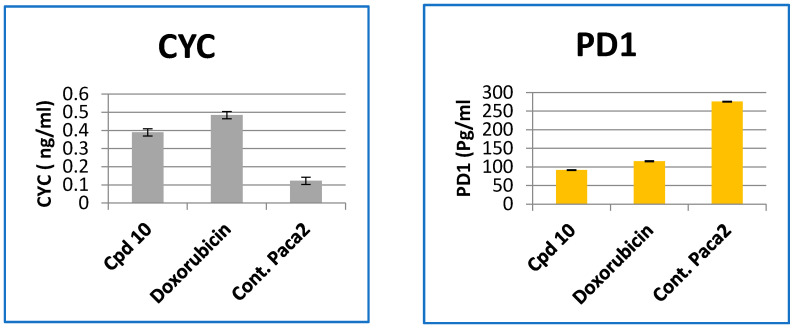
Casp3, Casp7, Cytochrome c and PD1 protein concentration in Paca-2 cells after treatment with compound **10** for 24 h.

Treating Paca-2 cells with the IC_50_ of compound **10** (10.7 μM) for 24 h, elevated the caspase-7 protein level by 4.3-fold from 0.555 ng/mL in control Paca-2 cells to 2.363 ng/mL in treated cells. Additionally, compound **10** upregulated caspase-3 protein level inside treated Paca-2 cells by 2.8-fold from 95.04 Pg/mL to 266.8 Pg/mL, as shown in Figure 7. Compound **10** increased the cytochrome-c release from the mitochondria into the cytosol of treated Paca-2 from 0.123 ng/mL to 0.395 ng/mL. Additionally, compound **10** had a potent inhibitory effect on PD-1 protein, as it downregulated the PD-1 level from 275.6 Pg/mL in untreated control Paca-2 to 91.4 Pg/mL in compound **10**-treated Paca-2 cells.

### 2.3. Molecular Docking Study

Based upon the kinase inhibitory results of the targets **10**, **13** and **15**, the docking simulation study was conducted in attempt to provide a correlation between their activities and the possible binding modes and orientations within the binding sites of EGFR, VEGFR-2 and CDK-2/cyclin A2 kinases. The docking process was applied using MOE-Dock (Molecular Operating Environment) software version 2014.0901 [68,69,70] and validated through re-docking of the native ligands erlotinib, sorafenib and roscovitine within the active sites of EGFR, VEGFR-2 and CDK-2/cyclin A2 (PDB codes: 1M17, 4ASD and 3DDQ, respectively) [65,71,72] giving energy scores of −12.10, −11.59 and −11.25 kcal/mol with small RMSD values of 0.88, 1.20 and 0.77 Å, respectively, between the co-crystallized ligands and their docked poses.

**Figure 8:**
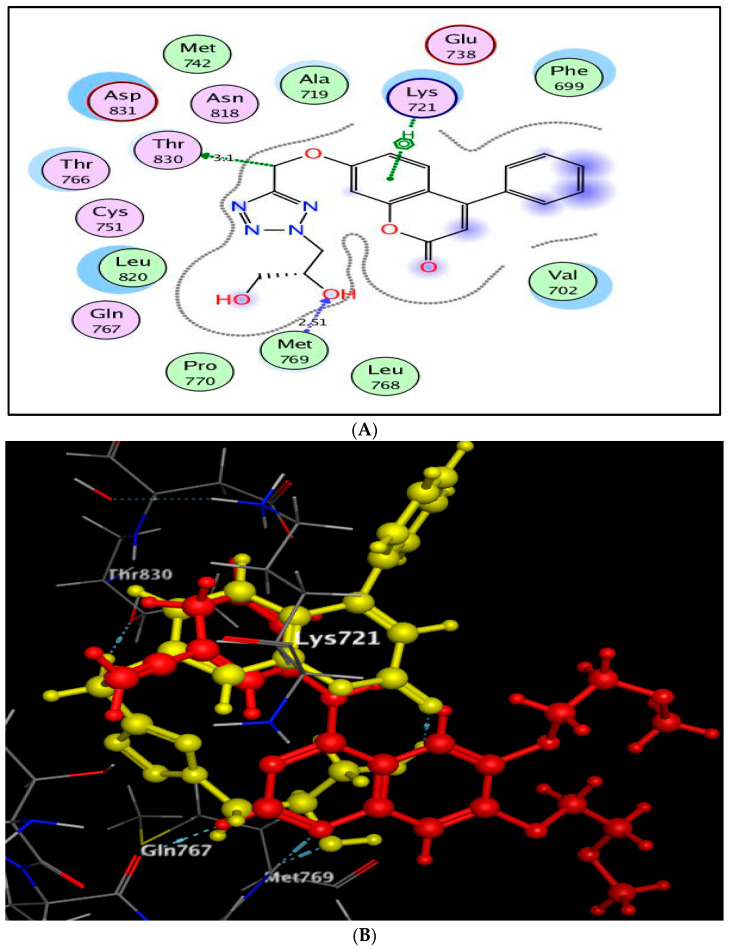
(**A**) view illustrates the 2D binding features of the coumarin target, **10**, while (**B**) view illustrates the 3D superimposition between native ligand erlotinib (red) and the target, **10** (yellow) within the active site of EGFR (PDB code: 1M17), respectively.

**Figure 9:**
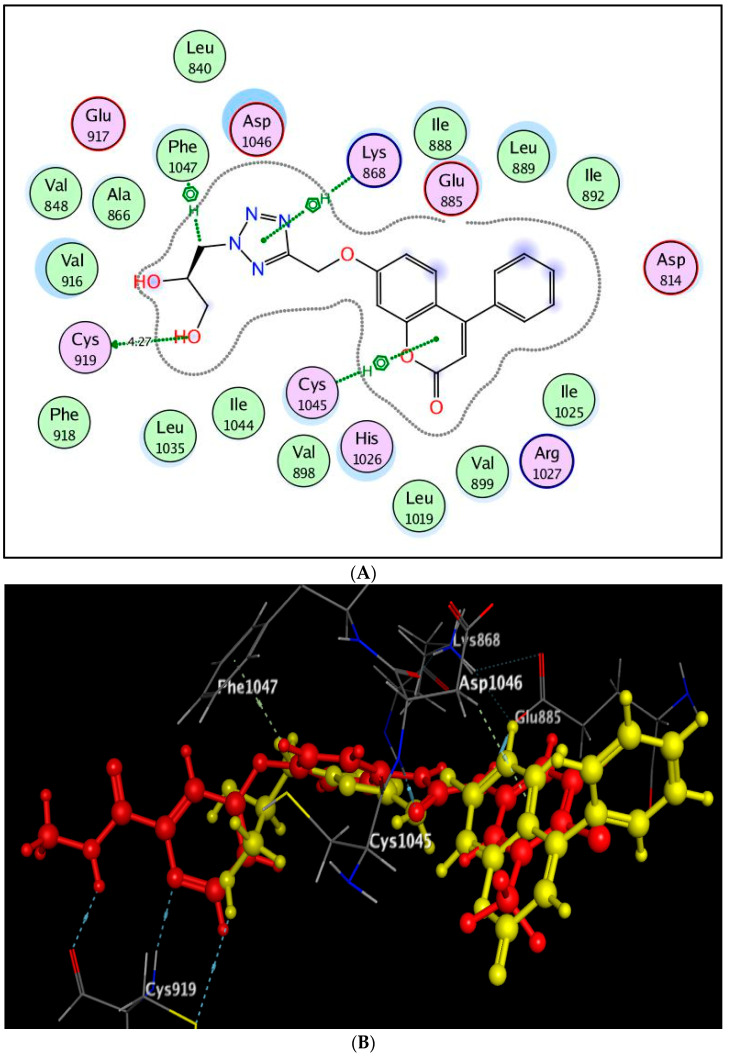
(**A**) view illustrates the 2D binding features of the coumarin target, **10**, while (**B**) view illustrates the 3D superimposition between native ligand sorafenib (red) and the target, **10** (yellow) within the active site of VEGFR-2 (PDB code: 4ASD), respectively.

**Figure 10:**
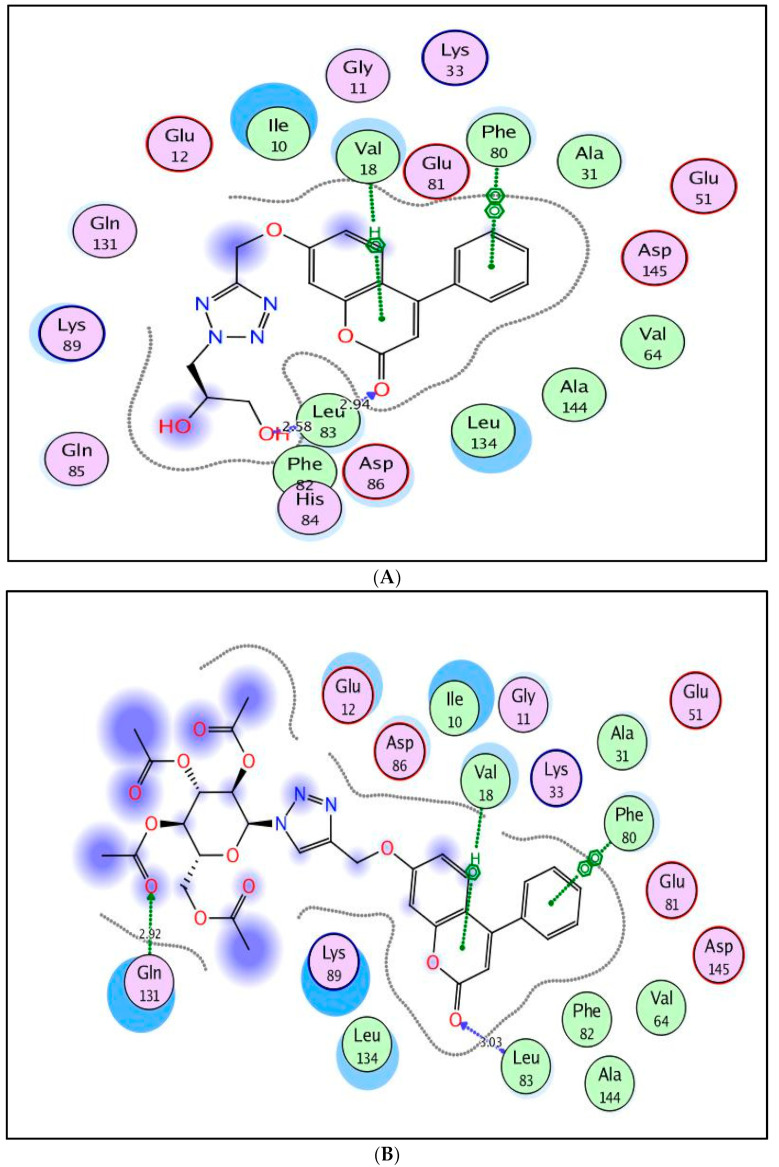

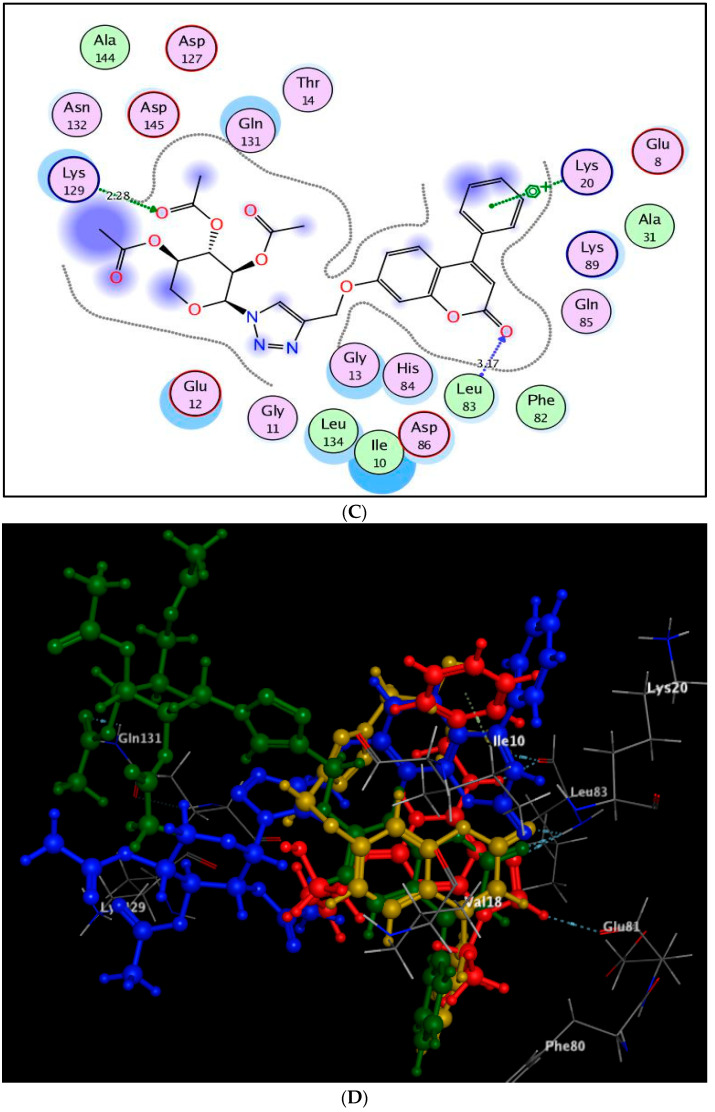
(**A**–**C**) views illustrates the 2D binding features of the coumarin targets, **10**, **13** and **15**, respectively while **(D**) view illustrates the 3D superimposition between native ligand roscovitine (red) and the targets, **10** (yellow), **13** (green) and **15** (blue) within the active site of CDK-2/cyclin A2 (PDB code: 3DDQ), respectively.

The existence of hydroxyl group of 2,3-dihydroxypropyl fragment in compound **10** facilitated its binding with the key amino acids of the screened receptors through H-bonding, **Met768** in EGFR, **Cys919** in VEGFR-2 and **Leu83** in CDK-2 (distance: 2.51, 4.27 and 2.58 Å, respectively) resembling the reference ligands and confirmed with good energy scores of −13.22, −11.10 and −11.30 kcal/mol, respectively and the superimposition figures (Figure 8A, Figure 9B and Figure 10D). 

Furthermore, the compound **10** potentiated its binding with EGFR through H-bonding between the oxomethylene carbon and **Thr830** (distance: 3.10 Å), and arene-cation interaction between the coumarin scaffold and **Lys721** (Figure 8A). While with VEGFR-2 it afforded three arene-cation interactions between coumarin, tetrazole and C-1 of propyl group with **Cys1045**, **Lys868** and **Phe1047**, respectively (Figure 9A). The flexibility of compound **10** within CDK-2 gave the chance for further hydrogen bonding between the carbonyl oxygen and the key amino acid **Leu83** (distance: 2.94 Å) (Figure 10A). Additionally, the binding with CDK-2 was supported by arene-cation interaction between coumarin and **Val18**, and another arene-arene interaction between the phenyl moiety and **Phe80**. 

By inspection of Figure 10, it was noted that the carbonyl oxygen of coumarin moiety in the compounds **10**, **13** and **15** played an essential role in fitting within CDK-2 through H-bond formation with the key amino acid **Leu83** (distance: 2.94, 3.03 and 3.37 Å, respectively). Moreover, the acetylated oxygens exhibited H-bonding with **Gln131** in compound **13** (distance: 2.92 Å) and with **Lys129** in compound **15** (distance: 2.28 Å). Both derivatives gave promising energy scores of −10.76 and −10.85 kcal/mol, respectively.

As a concluding point and regarding previous superimposition figures, it was noted that the presence of 2,3-dihydroxypropyl fragment (small size) in compound **10** boosted good fitting and binding with the key amino acids within the active sites of EGFR, VEGFR-2 and CDK-2/cyclin A2, compared to the other derivatives **13** and **15,** bearing large glycoside moieties. Additionally, the existence of the coumarin nucleus in the screened derivatives **10**, **13** and **15** has a vital role in fixation within the active site of CDK-2. 

## 3. Experimental

### 3.1. Chemistry

Chemicals were bought from Merck and Sigma-Aldrich and used without further purification. TLC was performed using aluminium plates pre-coated with silica gel 60 or 60 F254 (Merck) and visualized by iodine or UV light (254 nm). Melting points were determined on a Böetius PHMK (VebAnalytik Dresden) apparatus. The NMR spectra were recorded on a Varian Gemini 300 and Bruker DRX 400 spectrometer at 25 °C, unless otherwise stated. ^1^H- and ^13^C-NMR signals were referenced to TMS and the solvent shift ((CD_3_)_2_SO δH 2.50 and δC 39.5). Coupling constants are given in Hz and without signs. The IR-spectra were recorded by a Nexus 670 FT-IR FT-Raman spectrometer using KBr disc. IR, Ms spectra and the elemental analyses were performed at Micro-Analytical Laboratory, Central Services Laboratory, Faculty of Science, Cairo University, Egypt.

*2-((2-Oxo-4-phenyl-2H-chromen-7-yl)oxy)acetonitrile* (**2**). To a solution of 7-hydroxy-4-phenyl-2*H*-chromen-2-one (**1**) (2.77g, 10 mmole) in acetone (30 mL) was added anhydrous potassium carbonate (1.38 g, 10 mmole) and the mixture was stirred at room temperature for 1 h. 2-Chloroacetonitrile (0.74 mL 10 mmole) was added, and the reaction mixture was heated at reflux temperature for 8 h and then filtered. The filtrate was evaporated and the remaining solid was collected and recrystallized from ethanol to give compounds **2**.

Yield: 94%; mp 183–184 °C; IR (KBr) cm^−1^, ν: 3068 (C-H), 2261 (CN), 1705 (C=O); ^1^H NMR (DMSO-d_6_, δ, ppm): 5.30 (s, 2H, OCH_2_), 6.30 (s, 1H, coumarin-H^3^), 7.02–7.06 (m, 2H, Ar-H), 7.30 (d, 1H, *J* = 8.4 Hz, Ar-H), 7.40 (d, 1H, *J* = 8.4 Hz, Ar-H), 7.69–7.75 (m, 4H, Ar-H). ^13^C NMR (DMSO-d_6_) δ/ppm: 54.5 (CH_2_), 102.8, 112.9, 112.9, 113.1, 113.8, 116.6, 128.7, 128.9, 129.4, 130.2, 135.3, 155.3, 155.7, 159.7 (Ar-C, coumarin-C^3^,C^4^), 160.2 (coumarin-C^2^), MS *m*/*z*: 277.02 (M^+^, 27.02%). Anal. calcd. for C_17_H_11_NO_3_: C, 73.64; H, 4.00; N, 5.05. Found: C, 73.58; H, 4.05; N, 5.11.

*7-((2H-Tetrazol-5-yl)methoxy)-4-phenyl-2H-chromen-2-one* (**3**). A mixture of 2-((2-oxo-4-phenyl-2*H*-chromen-7-yl)oxy)acetonitrile (**2**) (3.2 g, 10 mmole), sodium azide (0.65 g, 10 mmole) and ammoninm chloride (0.53 g, 10 mmole) in *N*,*N*-dimethylformamide (30 mL) was heated for 7 h at 120 °C. The solvent was removed under reduced pressure and the residue was dissolved in water (100 mL) and carefully acidified with conc. hydrochloric acid to P_H_ ≈ 2. The solution was cooled to 0–5 °C in an ice bath in a refrigerator overnight and the precipitated solid was filtered, washed with water and recrystallized from ethanol-water mixture (1:1) to give compound **3**.

Yield: 90%; mp 219–221 °C; IR (KBr) cm^−1^, ν: 3421 (NH), 3055 (C-H), 2921 (C-H), 1707 (C=O); ^1^H NMR (DMSO-d_6_, δ, ppm): 5.61 (s, 2H, OCH_2_), 6.25 (s, 1H, coumarin-H^3^), 7.00 –7.08 (m, 2H, Ar-H), 7.31 (d, 1H, *J* = 8.4 Hz, Ar-H), 7.42 (d, 1H, *J* = 8.4 Hz, Ar-H), 7.53 (m, 4H, Ar-H), 12.05 (br, 1H, NH); ^13^C NMR (DMSO-d_6_) δ/ppm: 60.4 (CH_2_), 102.8, 110.9, 112.5, 112.2, 113.4, 128.5, 128.9, 129.4, 130.4, 153.9, 155.8, 160.3 (Ar-C, coumarin-C^3^,C^4^, tetrazol-C^5^), 160.9 (coumarin-C^2^), MS *m*/*z*: 320.20 (M^+^, 21.82%). Anal. calcd. for C_17_H_12_N_4_O_3_: C, 63.75; H, 3.78; N, 17.49. Found: 63.64; H, 3.68; N, 17.58.

*4-Phenyl-7-(1-(O-actyl-β-d-glycopyranosyl)-2H-tetrazol-5-yl)methoxy)-2H-chromen-2-one* (**5**,**6**). *General procedure:* To a solution of the tetrazole derivative **3** (3.2 g, 10 mmole) in dry DMF (30 mL) was added anhydrous potassium carbonate (1.38 g,10 mmole) and the mixture was stirred at room temperature for 1 h. 2,3,4,6-Tetra-*O*-acetyl-d-galctopyranosyl bromide or 2,3,4-tri-*O*-acety-d-xylopyranosyl bromide **4a**,**b** (10 mmole), dissolved in a least amount of DMF, was added portion wise and the mixture was stirred at room temperature until the reaction was complete, as assessed by TLC (6–7 h). The solvent was evaporated under reduced pressure and the residue was washed with a little amount of distilled water to remove the formed inorganic salt, then filtered and dried to give glycoside **5** and **6**, respectively.

*4-Phenyl-7-((1-(2,3,4,6-tetra-O-actyl-β-d-galactopyranosyl)-2H-tetrazol-5-yl)methoxy)-2H-chromen-2-one* (**5**). Yield: 65%; mp 122–124 °C; IR (KBr) cm^−1^, ν: 3055 (C-H), 2924 (C-H), 1710 (C=O), 1752 (C=O); ^1^H NMR (DMSO-d_6_) δ/ppm: 1.92, 1.96, 2.00, 2.01 (4s, 12H, 4CH_3_), 3.94–4.03 (dd, 1H, *J* = 2.8, *J* = 11.4 Hz, H-6′), 4.08- 4.14 (dd, 1H, *J* = 11.4, *J* = 3.8 Hz, H-6′′), 4.59–4.66 (m, 1H, H-5′), 5.23–5.30 (dd, 1H, *J* = 8.6, *J* = 9.2 Hz, H-4′), 5.42–5.48 (dd, 1H, *J* = 9.2, *J* = 10.2 Hz, H-2′), 5.59 (s, 2H, OCH_2_), 5.67–5.74 (t, 1H, *J* = 9.2 Hz, H-3′), 6.27 (d, 1H, *J* = 10.2 Hz, H-1′), 6.35 (s, 1H, coumarin-H^3^), 7.14 –7.21 (m, 2H, Ar-H), 7.33 (d, 1H, *J* = 8.4 Hz, Ar-H), 7.42 (d, 1H, *J* = 8.4 Hz, Ar-H), 7.80–7.93 (m, 4H, Ar-H, NH); ^13^C NMR (DMSO-d_6_) δ/ppm: 20.8, 20.9, 21.0, 21.1 (4CH_3_), 61.9 (C-6′), 67.7 (C-4′), 70.6 (C-2′), 73.9 (CH_2_), 76.5 (C-3′), 77.5 (C-5′), 97.6 (C-1′), 102.7, 110.6, 113.0, 113.3, 119.9. 128.5, 128.7, 128.8, 129.1, 153.3, 135.5, 159.8, 160.4 (Ar-C, coumarin-C^3^,C^4^, tetrazol-C^5^), 161.8 (coumarin-C^2^), 170.1, 170.2, 170.4, 170.5 (4C=O). Anal. calcd. for C_31_H_30_N_4_O_12_: C, 57.23; H, 4.65; N, 8.61. Found: C, 57.35; H, 4.60; N, 8.69.

*4-Phenyl-7-((1-(2,3,4-tri-O-acetyl-β-d-xylopyranosyl)-2H-tetrazol-5-yl)methoxy)-2H-chromen-2-one* (**6**). Yield: 70%; mp 125–126 °C; IR (KBr) cm^−1^, ν: 3059 (C-H), 2929 (C-H), 1712 (C=O), 1756 (C=O); ^1^H NMR (DMSO-d_6_) δ/ppm: 1.96, 2.00, 2.01 (3s, 9H, 3CH_3_), 3.88–3.91 (dd, 1H, *J* = 2.8, *J* = 10.8 Hz, H-5′), 3.95- 3.99 (dd, 1H, *J* = 10.8, *J* = 3.6 Hz, H-5′′), 5.34–5.38 (m, 1H, H-4′), 5.56–5.59 (dd, 1H, *J* = 7.8, *J* = 9.8 Hz, H-2′), 5.60 (m, 3H, H-3′, OCH_2_), 6.33 (d, 1H, *J* = 10.2 Hz, H-1′), 7.13 –7.20 (m, 3H, Ar-H, coumarin-H^3^), 7.32 (d, 1H, *J* = 8.4 Hz, Ar-H), 7.44 (d, 1H, *J* = 8.4 Hz, Ar-H), 7.82–7.97 (m, 4H, Ar-H, NH); ^13^C NMR (DMSO-d_6_) δ/ppm: 20.5, 20.6, 20.8 (3CH_3_), 62.5 (C-5′), 67.9 (C-4′), 70.7 (C-2′), 73.9 (CH_2_), 76.6 (C-3′), 97.9 (C-1′), 102.8, 110.6, 113.2, 113.5, 119.9. 128.5, 128.8, 128.7, 128.9, 129.1, 153.4, 135.5, 159.9, 160.4 (Ar-C, coumarin-C^3^,C^4^, tetrazol-C^5^), 161.9 (coumarin-C^2^), 170.3, 170.4, 170.6 (4C=O). Anal. calcd. for C_28_H_26_N_4_O_10_: C, 58.13; H, 4.53; N, 9.68. Found: C, 58.29; H, 4.48; N, 9.74.

*4-Phenyl-7-((1-(d-glycopyranosyl)-)-2H-tetrazol-5-yl)methoxy)-2H-chromen-2-one* (**7**,**8**). *General procedure:* A solution of the acetylated glycoside derivatives (**5**,**6**) (0.5 g) in saturated methanolic ammonia (20 mL) was stirred at room temperature for 7 h. After completion of the deacetylation reaction as indicated by TLC (petroleum ether-hexane, 2:1), the solvent was evaporated under reduced pressure at 40 °C to give a residue, which was triturated with diethyl ether (25 mL) with stirring to afford a solid, which was filtered, dried and crystallized from cold ethanol.

*4-Phenyl-7-((1-(β-d-galactopyranosyl)-)-2H-tetrazol-5-yl)methoxy)-2H-chromen-2-one* (**7**). Yield: 60%; mp 204–205 °C; IR (KBr) cm^−1^, ν: 3425–3385 (OH), 3072 (C-H), 2920 (C-H), 1697 (C=O); ^1^H NMR (DMSO-d_6_) δ/ppm: 3.49–3.58 (m, 3H, H-6′,6′′), 3.62–3.66 (m, 1H, H-5′), 4.16–4.29 (m, 1H, H-4′), 4.54–4.68 (dd, 1H, *J* = 7.8, *J* = 9.8 Hz, H-2′,), 4.95–5.03 (m, 3H, H-3′, 2OH), 5.24–5.29 (m, 2H, 2OH), 5.53 (s, 2H, OCH_2_), 5.83 (d, 1H, *J* = 9.8 Hz, H-1′), 6.26 (s, 1H, coumarin-H^3^), 7.12 –7.19 (m, 2H, Ar-H), 7.31 (d, 1H, *J* = 8.4 Hz, Ar-H), 7.44 (d, 1H, *J* = 8.4 Hz, Ar-H), 7.65–7.74 (m, 4H, Ar-H); ^13^C NMR (DMSO-d_6_) δ/ppm: 60.9 (C-6′), 68.7 (C-4′), 71.3 (C-2′), 73.8 (CH_2_), 73.8 (C-3′), 81.6 (C-5′), 100.0 (C-1′), 104.2, 112.4, 112.4, 114.4, 128.3. 128.9, 129.0, 129.1, 129.4, 129.5, 155.6, 155.7, 160.5 (Ar-C, coumarin-C^3^,C^4^, tetrazol-C_5_), 161.0 (coumarin-C_2_). Anal. calcd. for C_23_H_22_N_4_O_8_: C, 57.26; H, 4.60; N, 11.61. Found: C, 57.12; H, 4.68; N, 11.52.

*4-Phenyl-7-((1-(β-d-Xylopyranosyl)-)-2H-tetrazol-5-yl)methoxy)-2H-chromen-2-one* (**8**). Yield: 75%; mp 195–196 °C; IR (KBr) cm^−1^, ν: 3435–3415 (OH), 30,055 (C-H), 2923 (C-H), 1710 (C=O); ^1^H NMR (DMSO-d_6_) δ/ppm: 3.33–3.39 (m, 2H, H-5′,5′′), 3.88–3.97 (m, 2H, H-4′, H-2′), 4.45–4.55 (m, 2H, H-3′, OH), 4.80–4.83 (m, 1H, OH), 5.48–5.52 (m, 3H, OH, CH_2_), 5.71 (d, 1H, *J* = 9.8 Hz, H-1′), 6.21 (1H, s, coumarin-H^3^), 7.15–7.21 (m, 2H, Ar-H), 7.32 (d, 1H, *J* = 8.4 Hz, Ar-H), 7.49 (d, 1H, *J* = 8.4 Hz, Ar-H), 7.83–7.90 (m, 4H, Ar-H); ^13^C NMR (DMSO-d_6_) δ/ppm: 60.9 (C-5′), 68.9 (C-4′), 70.7 (C-2′), 73.8 (CH_2_), 73.9 (C-3′), 101.2 (C-1′), 104.1, 112.3, 112.4, 114.2, 128.3. 128.8, 129.0, 129.1, 129.3, 129.4, 155.6, 155.8, 160.6 (Ar-C, coumarin-C^3^,C^4^, tetrazol-C^5^), 161.9 (coumarin-C^2^). Anal. calcd. for C_22_H_20_N_4_O_7_: C, 58.41; H, 4.46; N, 12.38. Found: C, 58.59; H, 4.41; N, 12.30.

*Synthesis of N-substituted tetrazole compounds***9***and***10**. *General procedure:* To a solution of tetrazolyl-methoxy(coumarin) derivative **3** (3.2 g, 10 mmole) in dry acetonitrile (15 mL) was added anhydrous potassium carbonate (1.38 g, 10 mmole) and the mixture was stirred at room temperature for 1 h. 2-(2-chloroethoxy)ethan-1-ol or 3-chloropropane-1,2-diol (10 mmole) was added and stirring was continued for 18 h at 70 °C and then filtered. The solvent was evaporated, and the remaining solid material was recrystallized from ethanol to give compounds **9** or **10**, respectively. 

*7-((2-(2-(2-Hydroxyethoxy)ethyl)-2H-tetrazol-5-yl)methoxy)-4-phenyl-2H-chromen-2-one* (**9**). Yield: 65%; Pale yellow foam; IR (KBr) cm^−1^, ν: 3431 (OH), 3067 (C-H), 2924 (C-H), 1720 (C=O); ^1^H NMR (CDCl_3_) δ/ppm 3.56 (t, 2H, *J* = 6.8 Hz, CH_2_), 3.73–3.76 (m, 2H, CH_2_), 4.05 (t, 2H, *J* = 6.8 Hz, CH_2_), 4.19 (t, 2H, *J* = 6.6 Hz, CH_2_), 4.81–4.83 (t, 1H, *J* = 6.8 Hz, OH), 5.38 (s, 2H, CH_2_), 6.19 (s, 1H, coumarin-H^3^), 7.09–7.15 (m, 2H, Ar-H), 7.33 (d, 1H, *J* = 8.4 Hz, Ar-H), 7.50 (d, 1H, *J* = 8.4 Hz, Ar-H), 7.85–7.93 (m, 4H, Ar-H); ^13^C NMR (DMSO-d_6_) δ/ppm: 63.4 (CH_2_), 63.5 (CH_2_), 72.2 (CH_2_), 72.4 (CH_2_), 72.5 (CH_2_), 108.1, 112.4, 122.4, 123.9. 124.1, 124.2, 124.9, 125.00, 125.2, 130.7, 157.2,157,0, 161.00, 161.1 (Ar-C, coumarin-C^3^,C^4^, coumarin-C^2^). Anal. calcd. for C_21_H_20_N_4_O_5_: C, 61.76; H, 4.94; N, 13.72. Found: C, 61.51; H, 5.03; N, 13.61.

*7-((2-(2,3-Dihydroxypropyl)-2H-tetrazol-5-yl)methoxy)-4-phenyl-2H-chromen-2-one* (**10**). Yield: 64%, IR (KBr) cm^−1^, ν: 3426 (OH), 3045 (C-H), 2929 (C-H), 1711 (C=O); ^1^H NMR (CDCl_3_) δ/ppm: 3.55–3.58 (m, 2H, CH_2_), 3.67–370 (m, 2H, C*H*OH, N-C*H*_a_), 3.90 (dd, 1H, *J* = 7.8 Hz, N-C*H_b_*), 4.88 (t, 1H, *J* = 6.6 Hz, OH), 5.33 (s, 2H, OCH_2_) 5.36–5.40 (m, 1H, OH), 6.21 (s, 1H, coumarin-H^3^), 7.17–7.24 (m, 2H, Ar-H), 7.32 (d, 1H, *J* = 8.4 Hz, Ar-H), 7.52 (d, 1H, *J* = 8.4 Hz, Ar-H), 7.83–7.93 (m, 4H, Ar-H). Anal. calcd. for C_20_H_18_N_4_O_5_: C, 60.91; H, 4.60; N, 14.21. Found: C, 60.80; H, 4.66; N, 14.09.

*4-Phenyl-7-(prop-2-yn-1-yloxy)-2H-chromen-2-one* (**11**). To a solution of 7-hydroxy-4-phenyl-2*H*-chromen-2-one (**1**) (2.77g, 10 mmole) in acetone (30 mL) was added anhydrous potassium carbonate (1.38 mL, 10 mmole) and the mixture was stirred at room temperature for 1 h. 3-Bromo-1-propyne (1.1 g, 10 mmole) was added and the reaction mixture was heated at reflux temperature for 8 h and then filtered. The solvent was evaporated, and the remaining precipitate was washed with water and filtered, then dried and recrystallized from ethanol to give compound **11**.

Yield: 95%; mp 116–117 °C; IR (KBr) cm^−1^, ν: 3258 (acetylenic-CH), 3068 (C-H), 2929 (C-H), 2126 (C-C alkyne), 1720 (C=O); ^1^H NMR (CDCl_3_) δ/ppm: 2.75 (s, 1H, CH), 4.76 (s, 2H, CH_2_), 6.23 (s, 1H, coumarin-H^3^), 7.00–7.18 (m, 2H, Ar-H), 7.30 (d, 1H, *J* = 8.4 Hz, Ar-H), 7.49 (d, 1H, *J* = 8.4 Hz, Ar-H), 7.70–7.85 (m, 4H, Ar-H). MS *m*/*z*: 277.15 (M^+^ + 1, 21.86%),276.20 (M^+^, 100%). Anal. calcd. for C_18_H_12_O_3_: C, 78.25; H, 4.38 Found: C, 78.37; H, 4.31.

*4-Phenyl-7-((1-(O-acetyl-β-d-sugarpyranosyl)-1H-1,2,3-triazol-4-yl)methoxy)-2H-chromen-2-one* (**13**–**15**). To a solution of the alkyne substrate **11** (2.0 mmol) in *n*-butanol (25 mL), the glycopyranosyl azide derivative **12a-c**, namely, 2,3,4,6-tetra-*O*-acetyl-d-glucopyranosyl, 2,3,4,6-tri-*O*-acetyl-d-galctopyranosyl or 2,3,4-tri-*O*-acetyl-d-xylopyranosyl azide (2 mmol) was added. Sodium ascorbate (0.4 mmol, 0.08 g) dissolved in water (2 mL) and few drops of diisopropylethylamine (DIPEA) followed by CuSO_4_.5H_2_O (0.4 mmol, 0.11 g) dssolved in water (2 mL) were then added separately. The mixture was stirred at room temperature overnight [(TLC judged; pet. ether: ethyl acetate (4:1)]. Ethyl acetate was then added, and the organic layer was then speared after shaking the mixture twice for five minutes, followed by washing with water (2 × 20 mL)_._ The organic layers were then collected then dried (Na_2_SO_4_) and evaporated. Further purification by column chromatography [hexane: ethyl acetate (5:1) as eluent] gave the title products **13**–**15**, respectively.

*4-Phenyl-7-((1-(2,3,4,6-Tetra-O-acetyl-β-d-glucopyranosyl)-1H-1,2,3-triazol-4-yl)methoxy)-2H-chromen-2-one* (**13**). Yield: 71%; mp 131–132 °C; IR (KBr) cm^−1^, ν: 3070 (C-H), 2924 (C-H), 1720 (C=O), 1749 C=O); ^1^H NMR (DMSO-d_6_) δ/ppm: 1.98, 1.99, 2.03, 2.04 (4s, 12H, 4CH_3_), 3.62–3.77 (dd, 1H, *J* = 3.4, *J* = 10.8 Hz, H-6′), 4.16- 4.25 (dd, 1H, *J* = 10.8, *J* = 3.8 Hz, H-6′′), 4.49–4.54 (m, 1H, H-5′), 5.23–5.29 (m, 3H, CH_2_, H-4′), 5.23–5.26 (dd, 1H, *J* = 7.8, *J* = 9.8 Hz, H- H-2′), 5.55 (t, 1H, *J* = 7.8 Hz, H-3′), 6.12 (s, 1H, coumarin-H^3^), 6.19 (d, 1H, *J* = 9.8 Hz, H-1′), 7.09–7.17 (m, 2H, Ar-H), 7.32 (d, 1H, *J* = 8.4 Hz, Ar-H), 7.50 (d, 1H, *J* = 8.4 Hz, Ar-H), 7.68–7.78 (m, 5H, Ar-H). Anal. calcd. for C_32_H_31_N_3_O_12_: C, 59.17; H, 4.81; N, 6.47. Found: C, 59.02; H, 4.86; N, 6.41.

*4-Phenyl-7-((1-(2,3,4,6-tetra-O-acetyl-β-d-galactopyranosyl)-1H-1,2,3-triazol-4-yl)methoxy)-2H-chromen-2-one* (**14**). Yield: 69%; m.p. 93–94 °C; IR (KBr) cm^−1^, ν: 3055 (C-H), 2922 (C-H), 1715 (C=O), 1747 (C=O); ^1^H NMR (DMSO-d_6_) δ/ppm: 1.92, 1.95, 1.97, 2.01 (4s, 12H, 4CH_3_), 3.94–3.99 (dd, 1H, *J* = 2.8, *J* = 10.8 Hz, H-6′), 4.14- 4.20 (dd, 1H, *J* = 10.8, *J* = 7.8 Hz, H-6′′), 4.87–4.98 (m, 1H, H-5′), 5.28 (s, 2H, OCH_2_), 5.32–5.34 (dd, 1H, *J* = 7.8, *J* = 8.8 Hz, H-4′), 5.43–5.48 (dd, 1H, *J* = 8.8, *J* = 9.8 Hz, H-2′), 5.58–5.64 (t, 1H, *J* = 8.8 Hz, H-3′), 6.19 (1H, s, coumarin-H^3^), 6.30 (d, 1H, *J* = 9.8 Hz, H-1′), 7.09–7.16 (m, 2H, Ar-H), 7.32 (d, 1H, *J* = 8.4 Hz, Ar-H), 7.51 (d, 1H, *J* = 8.4 Hz, Ar-H), 7.66–7.78 (m, 5H, Ar-H). ^13^C NMR (DMSO-d_6_) δ/ppm: 19.2, 20.4, 20.9, 21.0 (4CH_3_), 62.1 (C-6′), 68.3 (C-4′), 67.8 (CH_2_), 70.9 (C-2′), 73.5 (C-3′), 79.7 (C-5′), 99.0 (C-1′), 102.6, 102.7, 112.0, 112.6, 113.5. 128.3, 128.9, 129.4, 130.2, 135.4, 143.1, 155.6, 155.9, 160.5 (Ar-C, coumarin-C^3^,C^4^, triazole-C^4^,C^5^), 161.5 (coumarin-C^2^), 170.0, 170.4, 170.5 (4C=O). Anal. calcd. for C_32_H_31_N_3_O_12_: C, 59.17; H, 4.81; N, 6.47. Found: C, 59.29; H, 4.88; N, 6.38.

*4-Phenyl-7-((1-(2,3,4-tria-O-acetyl-β-d-xylopyranosyl)-1H-1,2,3-triazol-4-yl)methoxy)-2H-chromen-2-one* (**15**). Yield: 65%; mp 89–90 °C; IR (KBr) cm^−1^, ν: 3058 (C-H), 2923 (C-H), 1718 (C=O), 1745 (C=O); ^1^H NMR (DMSO-d_6_) δ/ppm: 1.96, 1.99, 2.03 (3s, 9H, 3CH_3_), 3.74–3.76 (dd, 1H, *J* = 3.8, *J* = 10.6 Hz, H-5′), 3.78–3.80 (dd, 1H, *J* = 10.6, *J* = 7.8 Hz, H-5′′), 4.01–4.11 (dd, 1H, *J* = 7.8, *J* = 8.8 Hz, H-4′), 5.25 (s, 2H, CH_2_), 5.48–5.55 (m, 1H, H-2′), 5.81–5.95 (dd, 1H, *J* = 8.8, *J* = 9.2 Hz, H-3′), 6.38 (d, 1H, *J* = 10.2 Hz, H-1′), 7.02–7.12 (m, 3H, coumarine-H^3^, Ar-H), 7.33 (d, 1H, *J* = 8.4 Hz, Ar-H), 7.50 (d, 1H, *J* = 8.4 Hz, Ar-H), 7.67–7.92 (m, 5H, Ar-H). Anal. calcd. for C_29_H_27_N_3_O_10_: C, 60.31; H, 4.71; N, 7.28. Found: C, 60.42; H, 4.65; N, 7.39.

*4-Phenyl-7-((1-(β-d-glycopyranosyl)-1H-1,2,3-triazol-4-yl)methoxy)-2H-chromen-2-one* (**16**–**18**). The *O*-acetylated-1,2,3-triazole glycosidic compounds **13**–**15** (5 mmol) was added portion wise while stirring to dry methanol saturated with gaseous ammonia (20 mL) at 0 °C. The reaction mixture was stirred at room temperature for a further 7 h. After completion of the deacetylation process (TLC: petroleum ether-hexane, 2:1), the solvent was evaporated under reduced pressure at 40 °C to give a residue, which, upon treatment with cold diethyl ether (25 mL), afforded a solid product that was filtered and dried. 

*4-Phenyl-7-((1-(β-d-glucopyranosyl)-1H-1,2,3-triazol-4-yl)methoxy)-2H-chromen-2-one* (**16**). Yield: 70%; mp 180–182 °C; IR (KBr) cm^−1^, ν: 3427–3400 (OH), 3070 (C-H), 2922 (C-H), 1716 (C=O); ^1^H NMR (DMSO-d_6_) δ/ppm: 3.50–3.55 (m, 2H, H-6′,6′′), 3.60–3.66 (m, 2H, H-5′, H-4′), 3.72–3.75 (m, 2H, H-3′, H-2′,), 4.09–4.12 (m, 1H, OH), 5.06–5.17 (m, 2H, 2OH), 5.22–5.28 (m, 3H, CH_2,_ OH), 5.77 (d, 1H, *J* = 9.8 Hz, H-1′), 6.21 (s, 1H, coumarin-H^3^), 7.10–7.18 (m, 2H, Ar-H), 7.37 (d, 1H, *J* = 8.4 Hz, Ar-H), 7.52 (d, 1H, *J* = 8.4 Hz, Ar-H), 7.81–7.98 (m, 5H, Ar-H). ^13^C NMR (DMSO-d_6_) δ/ppm: 62.1 (C-6′), 79.1 (CH_2_), 72.6 (C-4′), 77.5 (C-2′), 80.53 (C-3′), 88.1 (C-5′), 102.0 (C-1′), 112.1, 112.6, 113.4, 113.4, 124.7, 128.9, 129.4, 130.2, 135.4, 142.4, 155.6, 155.9, 160.5 (Ar-C, coumarin-C^3^,C^4^, triazole-C^4^,C^5^), 161.7 (coumarin-C^2^) Anal. calcd. for C_24_H_23_N_3_O_8_: C, 59.87; H, 4.82; N, 8.73. Found: 59.71; H, 4.75; N, 8.82.

*4-Phenyl-7-((1-(β-d-galactopyranosyl)-1H-1,2,3-triazol-4-yl)methoxy)-2H-chromen-2-one* (**17**). Yield: 71%; mp 185–186 °C; IR (KBr) cm^−1^, ν: 3422–3390 (OH), 3055 (C-H), 2924 (C-H), 1713 (C=O); ^1^H NMR (DMSO-d_6_) δ/ppm: 3.52–3.58 (m, 2H, H-6′,6′′), 3.63–3.69 (m, 2H, H-5′, H-4′), 3.73–378 (m, 2H, H-3′, H-2′,), 4.08–4.11 (m, 1H, OH), 5.04–5.22 (m, 2H, 2OH), 5.25–5.31 (m, 3H, OH, OCH_2_), 5.79 (d, 1H, *J* = 10.2, H-1′), 6.21 (s, 1H, coumarin-H^3^), 7.15–7.22 (m, 2H, Ar-H), 7.39 (d, 1H, *J* = 8.4 Hz, Ar-H), 7.51 (d, 1H, *J* = 8.4 Hz, Ar-H), 7.73–7.90 (m, 5H, Ar-H).

^13^C NMR (DMSO-d_6_) δ/ppm: 62.3 (C-6′), 69.5 (CH_2_), 74.3 (C-4′), 78.9 (C-2′), 79.4.7 (C-3′), 88.7 (C-5′), 102.6 (C-1′), 102.8, 103.2, 112.0, 112.2, 113.4, 128.3, 128.9,129.3, 130.1, 135.4, 135.7, 155.6, 155.9, 160.4 (Ar-C, coumarin-C^3^,C^4^, triazole-C^4^,C^5^), 160.8 (coumarin-C^2^) Anal. calcd. for C_24_H_23_N_3_O_8_: C, 59.87; H, 4.82; N, 8.73. Found: 60.02; H, 4.89; N, 8.64.

*4-Phenyl-7-((1-(β-d-Xylopyranosyl)-1H-1,2,3-triazol-4-yl)methoxy)-2H-chromen-2-one* (**18**). Yield: 69%; mp 177–178 °C; IR (KBr) cm^−1^, ν: 3420–3395 (OH), 3048 (C-H), 2927 (C-H), 1711 (C=O); ^1^H NMR (DMSO-d_6_) δ/ppm: 3.74–3.79 (m, 2H, H-5′,5′′), 3.93–3.98 (m, 1H, H-4′), 4.08–4.12 (m, 1H, H-2′), 4.40–4.48 (dd, 1H, *J* = 7.6, *J* = 8.8 Hz, H-3′), 4.93–4.95 (m, 1H, OH), 5.18–5.31 (m, 4H, CH_2_, 2OH), 5.92 (d, 1H, *J* = 10.2 Hz, H-1′), 6.22 (1H, s, coumarin-H^3^), 7.11–7.19 (m, 2H, Ar-H), 7.37 (d, 1H, *J* = 8.4 Hz, Ar-H), 7.55 (d, 1H, *J* = 8.4 Hz, Ar-H), 7.74–7.91 (m, 5H, Ar-H). Anal. calcd. for C_23_H_21_N_3_O_7_: C, 61.19; H, 4.69; N, 9.31. Found: 61.27; H, 4.61; N, 9.42.

### 3.2. Biological Evaluation

#### 3.2.1. In Vitro Cytotoxicity

Human melanoma (A-375), melanoma (Mel-501), colon carcinoma (Caco-2), prostate cancer (PC-3) and pancreatic cancer (Paca-2) were obtained from Karolinska Centre, Department of Oncology and Pathology, Karolinska Institute and Hospital, Stockholm, Sweden. 

The cytotoxic screening of all synthesized derivatives **1**–**18** were assessed using the 3-[4,5- dimethyl-2-thiazolyl)-2,5-diphenyl-2 H-tetrazolium bromide (MTT) assay, following the previously reported method [61,62,63]. 

Following culturing for 10 days, the cells were seeded at a concentration of 10 × 10^3^ cells per well in case of PC-3, 20 × 10^3^ cells/well in a fresh complete growth medium in case of A-375, caco2, Paca-2 and Mel-501 at 37 °C for 24 h in water-jacketed carbon dioxide incubator. After 48 h incubation, the medium was aspirated and then 40 μL MTT salt (2.5 mg/mL) was added for a further 4 h. Then, 200 μL 10% sodium dodecyl sulphate (SDS) in deionized water was added to each well and incubated overnight at 37 °C. The absorbance was measured at 595 nm with reference 690 nm. Doxorubicin was used as the positive control and 0.5% DMSO was used as the negative control. The IC_50_ values were calculated, using a probit analysis, and by utilizing the SPSS computer program (SPSS for windows, statistical analysis software package/version 9/1989 SPSS Inc., Chicago, IL, USA).

#### 3.2.2. In Vitro Kinase Inhibitory Assessment against EGFR, VEGFR-2 and CDK-2

The targets with promising cytotoxicity **10**, **13** and **15** were evaluated for their in vitro inhibitory activity against EGFR, VEGFR-2 and CDK-2/cyclin A2 using erlotinib, sorafenib and roscovitine as references, respectively, following the previously mentioned procedure [5,64,65]. 

EGFR assay: The master mixture (6 μL 5X Kinase Buffer + 1 μL ATP (500 μM) + 1 μL 50 X PTK substrate + 17 μL water) was prepared, then 25 μL to every well was added along with 5 μL of inhibitor solution of each well labelled as “Test Inhibitor”. However, for the “Positive Control” and “Blank”, 5 μL of the same solution without inhibitor (Inhibitor buffer) was added. Then, 3 mL of 1X Kinase Buffer by mixing 600 μL of 5X Kinase Buffer with 2400 μL water was prepared. So, 3 mL of 1X Kinase Buffer became sufficient for 100 reactions. To the wells designated as “Blank”, 20 μL of 1X Kinase Buffer was added. EGFR enzyme on ice was thawed. Upon first thaw, briefly, the tube containing the enzyme was spun to recover the full content of the tube. The amount of EGFR required for the assay and dilute enzyme to 1 ng/μL with 1X Kinase Buffer was calculated. Moreover, the remaining undiluted enzyme in aliquots was stored at −80 °C. The reaction was initiated by adding 20 μL of diluted EGFR enzyme to the wells designated “Positive Control” and “Test Inhibitor Control”, after that it was incubated at 30 °C for 40 min. After the 40 min reaction, 50 μL of Kinase-Glo Max reagent was added to each well and the plate was covered with aluminium foil and incubated at room temperature for 15 min. Luminescence was measured using the microplate reader.

VEGFR-2 assay: Additionally, the effect of the most promising cytotoxic compounds **1** and **3c** on the level of VEGFR-2 in human breast cancer cell line MCF-7 was determined. The cells in culture medium were treated with 20 μL of IC_50_ values of the compounds dissolved in DMSO, then incubated for 24 h at 37 °C, in a humidified 5% CO_2_ atmosphere. The cells were harvested, and the homogenates were prepared in saline using a tight pestle homogenizer until complete cell disruption. The kit used a double-antibody sandwich enzyme-linked immunosorbent assay (ELISA) to determine the level of human VEGFR-2 in samples. A monoclonal antibody for VEGFR-2 was pre-coated onto 96-well plates. The test samples were added to the wells and a biotinylated detection polyclonal antibody from goat specific for VEGFR-2 was added, subsequently followed by washing with PBS buffer. Avidin–Biotin–Peroxidase complex was added, and the unbound conjugates were washed away with PBS buffer. HRP substrate TMB was used to visualize HRP enzymatic reaction. TMB was catalysed by HRP to produce a blue colour product that changed into yellow after the addition of acidic stop solution. The density of yellow colour is proportional to the human VEGFR-2 amount of the sample captured in the plate. The chroma of colour and the concentration of the human VEGFR-2 of the samples were positively correlated and the optical density was determined at 450 nm. The level of human VEGFR-2 in samples was calculated (pg/mL) as duplicate determinations from the standard curve. The % inhibition was calculated in comparison to control untreated cells.

Assay of CDK-2/cyclin A2 was achieved through ELISA using an affinity tag labelled capture antibody. Adding the samples and the standard to the wells was carried out after the addition of the antibody mix. After the incubation period, the unrestrained substance was discarded and the wells were washed. The added TMB (3,3′,5,5′-tetramethylbenzidine) substrate was prompted by Horseradish peroxidase (HRP) and blue coloration appeared. This reaction was stopped with the addition of stop solution to complete the change in colour from blue to yellow. The created signals were equivalent to the quantity of bound analyte and Robonik P2000 ELISA reader was used to record the intensity at a certain wavelength (450 nm). The concentrations of the screened compounds were calculated through the plotted curve.

#### 3.2.3. Cell Cycle Analysis and Apoptosis Detection of Compound **10**

Apoptosis investigation and cell cycle analysis were elucidated by flow cytometry [66]. Paca-2 cells were seeded at 8 × 10^4^ and incubated at 37 °C in 5% CO_2_ overnight. After treatment with the tested compound **10** for 24 h, cell pellets were collected and centrifuged (300× *g*, 5 min). For cell cycle analysis, cell pellets were fixed with 70% ethanol on ice for 15 min and collected again. The pellets were incubated with propidium iodide (PI) staining solution at room temperature for 1 h and analysed by a Gallios flow cytometer (Beckman Coulter, Brea, CA, USA). Apoptosis detection was carried out by FITC AnnexinV/PI commercial kit (Becton Dickenson, Franklin Lakes, NJ, USA) following the manufacturer’s protocol. The samples were analysed by fluorescence-activated cell sorting (FACS) with a Gallios flow cytometer (Beckman Coulter, Brea, CA, USA) within 1 h after staining. Data were analysed using Kaluza v 1.2 (Beckman Coulter, Brea, CA, USA).

#### 3.2.4. Effect of Compound **10** on the Levels of Caspase-3, 7, Cytochrome-c and PD1 Protein in Paca-2

The precoated micro-ELISA plate with an antibody specific to Caspase-3 was applied using the Human Caspase-3 ELISA Kit from Invitrogen INC. Catalog # KHO1091 (96 tests) (542 Flynn Road, Camarillo, CA, USA) according to the manufacturer’s instructions [67].

The micro-ELISA plate provided in this kit was pre-coated with a Caspase-7-specific antibody. A biotinylated Caspase-7 antibody and Avidin–horseradish peroxidase (HRP) conjugate was added. The excess components were removed. The substrate solution was added. Wells containing Caspase-7, biotinylated detection antibody, and Avidin-HRP conjugate appeared blue in colour. The colour turned yellow following the addition of sulphuric acid solution. The optical density (OD) was measured at a wavelength of 450 ± 2 nm.

Cytochrome-c specific antibodies were precoated onto 96-well plates. Cytochrome-c were analysed by enzyme-linked immunosorbent assays (ELISAs) using commercial kits (ab119521; Abcam, Cambridge, MA, USA). The optimal dilutions were determined and ELISAs were performed according to the manufacturer’s instructions.

Samples or standards were added to the wells Human PD-1 eliza kit (ab252360 Human PD-1, Abcam, Cambridge, MA, USA), followed by the antibody mix. After incubation, the wells were washed to remove unbound material. TMB Development Solution was added and during incubation was catalysed by HRP, generating blue coloration. This reaction was then stopped by the addition of Stop Solution, completing any colour change from blue to yellow. The optical density (OD) was measured at a wavelength of 450 ± 2 nm.

### 3.3. Molecular Docking Study

Molecular docking is a powerful computational process to rationalize gained biological data. Interactions between the newly synthesized target **10** with the highest inhibitory activity were examined within the active sites of EGFR, VEGFR-2 and CDK-2/cyclin A2 (PDB codes: 1M17, 4ASD and 3DDQ, respectively) [65,71,72] using MOE-Dock (Molecular Operating Environment) software version 2014.0901 [68,69,70]. Furthermore, the binding modes of the derivatives **13** and **15** were studied against CDK-2/cyclin A2. 

The 2D structure of the newly synthesized derivatives **10**, **13** and **15** were drawn through chem. Draw. The protonated 3D was employed using standard bond lengths and angles. Then, the geometry optimization and energy minimization were applied to obtain the Conf Search module in MOE, followed by saving of the MOE file for the upcoming docking process. The co-crystallized structures of EGFR, VEGFR-2 and CDK-2/cyclin A2 kinases with their ligands erlotinib, sorafenib and roscovitine were downloaded (PDB codes: 1M17, 4ASD and 3DDQ, respectively) from the protein data bank. All minimizations were performed using MOE until an RMSD gradient of 0.05 kcal∙mol^−1^Å^−1^ with MMFF94x force field and the partial charges was automatically calculated. The preparation of the enzyme structures was carried out for molecular docking using Protonate 3D protocol with the default options in MOE. London dG scoring function and the Triangle Matcher placement method were used in the docking protocol. At first, validation of the docking processes was established by docking the native ligands, followed by docking of the derivatives **10**, **13** and **15** within the ATP-binding sites after the elimination of the co-crystallized ligands.

## 4. Conclusions

The present work afforded the design and synthesis of a new set of triazole-coumarin-glycosyl hybrids and their tetrazole analogues. The antiproliferative activity of these derivatives were carried out against human Paca-2, Mel-501, PC-3, A-375 and Caco-2 cancer cell lines through colorimetric MTT assay and compared with the standard drug, doxorubicin. The promising coumarin derivatives **10**, **13** and **15** derivatives against Paca-2, Mel-501, PC-3 and A-375 cancer cell lines were further assessed for their inhibitory activities against EGFR, VEGFR-2 and CDK-2/cyclin A2 kinases to detect their mechanism of action. The coumarin-tetrazole **10** illustrated excellent broad inhibitory activity in comparison with the reference drugs, erlotinib, sorafenib and roscovitine, respectively. The molecular docking simulation was carried out to confirm these promising enzymatic results. Moreover, mechanistic studies found that the coumarin-tetrazole **10** arrested the cell cycle at pre-G 1 and S phases and induced apoptosis through upregulating casp-3, casp-7 and cytochrome-c proteins and downregulating PD-1 levels, in comparison with the untreated MCF-7 cells. Finally, this study may effectively promote further achievements in the field of discovering anticancer drug candidates.

## Figures and Tables

**Table 1:** In vitro cytotoxicity of the synthesized compounds **1**–**18** against human cancer Paca-2, Mel-501, PC-3, A-375 and Caco-2 cell lines at 100 µM.

Compound No.	% Inhibition				
	Paca-2	Mel-501	PC-3	A-375	Caco-2
**1**	79	96.2	55.7	95	40.5
**2**	44.1	35.6	10.5	6.5	-
**3**	55.6	48.4	12.2	55.3	-
**5**	56.9	68.4	15	23.1	-
**7**	21.3	22.3	0	13.8	-
**6**	72.1	71	9.1	45.8	-
**8**	96.5	98.1	98.1	95	97
**9**	94.7	97.7	95.9	84.1	99.5
**10**	99.9	95.8	99.2	98.2	99.6
**11**	58.1	44	16.7	9.1	0
**13**	97.6	98.5	95.6	96.9	99.6
**14**	94.9	77.1	80.2	96.8	89.8
**15**	95	97.8	98.2	94.7	99.3
**16**	96.9	99.1	95.8	97.5	99
**17**	96.3	98.6	97.3	98.5	95.6
**18**	96.9	98.5	91.4	97.9	99.4

(-) No activity detected.

**Table 2:** In vitro cytotoxic activity of the synthesized compounds **1**–**18** against human cancer Paca-2, Mel-501, PC-3, A-375 and Caco-2 cell lines.

Compound No.	IC_50_ (Mean ± SEM) (μM)				
	Paca-2	Mel-501	PC-3	A-375	Caco-2
**1**	62.3 ± 1.3	28.6 ± 0.4	---	12.9 ± 0.6	---
**5**	---	72.1 ± 1.2	---	---	---
**6**	60 ± 1.4	80.4 ± 2.1	---	---	---
**8**	23.8 ± 0.9	19.4 ± 0.4	48.2 ± 0.7	13.9 ± 0.2	52.5 ± 1.3
**9**	28.3 ± 0.7	30.2 ± 0.5	47.4 ± 1.4	15 ± 0.04	38.1 ± 0.4
**10**	10.7 ± 0.4	8.8 ± 0.2	25.4 ± 0.5	10.9 ± 0.1	31.9 ± 0.7
**13**	14.6 ± 1.1	16.7 ± 0.3	22.3 ± 0.6	14.5 ± 0.2	40.5 ± 0.9
**14**	33.4 ± 0.6	65.1 ± 1.2	51.3 ± 1.6	12.4 ± 0.3	70.2 ± 1.4
**15**	16.9 ± 0.9	4.1 ± 0.3	12.5 ± 0.9	9.9 ± 1	20.4 ± 0.6
**16**	23.8 ± 1.1	26.5 ± 0.7	36.4 ± 1.1	16.3 ± 0.6	48.1 ± 0.4
**17**	33.3 ± 0.5	35.8 ± 0.7	41 ± 2.1	39.6 ± 0.5	48.1 ± 0.8
**18**	36.4 ± 1.2	10 ± 0.1	37.6 ± 0.4	8.5 ± 0.3	61.9 ± 1.3
Doxorubicin	19.4 ± 0.5	3.5 ± 0.3	6.8 ± 0.2	4.5 ± 0.07	3.7 ± 0.2

IC_50_: Concentration of the synthesized derivative needed to inhibit the cell viability by 50%, SEM: Standard error mean; each value is the mean of three independent determinations, ---: not tested.

**Table 3:** Inhibitory assessment of the screened compounds **10**, **13** and **15** against EGFR, VEGFR-2 and CDK-2/cyclinA2 in comparison with erlotinib, sorafenib and roscovitine, respectively.

Compound No.	IC_50_ (Mean ± SEM) (µM)		
	EGFR	VEGFR-2	CDK-2/Cyclin A2
**Erlotinib**	0.13 ± 0.020	-	-
**Sorafenib**	-	1.050 ± 0.20	-
**Roscovitine**	-	-	0.37 ± 0.22
**10**	0.09 ± 0.21	0.86 ± 0.25	0.19 ± 0.05
**13**	66 ± 0.01	45 ± 0.10	0.50± 0.11
**15**	53 ± 0.15	40 ± 0.05	0.48± 0.15

IC_50_: Compound concentration necessary to inhibit the enzyme activity by 50%, SEM: Standard error mean; each value is the mean of three values, (-) not detected.

## Data Availability

Not applicable.

## Sample Availability

Samples of the compounds (**1**–**18**) are available from the authors.
